# Peripheral nerve injury recovery enhanced by ceftriaxone: a preclinical investigation

**DOI:** 10.1590/acb404625

**Published:** 2025-07-18

**Authors:** Berkin Gunar, Ejder Saylav Bora, Osman Mert Topkar, Özgür Baysal, Ziya Shammadli, Oytun Erbas

**Affiliations:** 1 Uşak Banaz State Hospital – Department of Orthopedics Surgery and Traumatology – Usak – Turkey.; 2 Marmara Unıversity – Faculty of Medicine – Department of Orthopedics Surgery and Traumatology – Istanbul – Turkey.; 3 Izmir Katip Çelebi University – Faculty of Medicine – Department of Emergency Medicine – Izmir – Turkey.; 4 Republican Clinical Hospital Named after G. G. Mirgasimov – Department of Traumatology and Orthopaedics Surgery – Baku – Azerbaijan.; 5 Biruni University – Faculty of Medicine – Biruni Research Center – Istanbul, Turkey.

**Keywords:** Nerve Growth Factors, Ceftriaxone, Experimental Study

## Abstract

**Purpose:**

To evaluate ceftriaxone’s potential in enhancing peripheral nerve injuries (PNIs) recovery.

**Methods:**

Thirty male Wistar rats were divided into control (no surgery), vehicle (sciatic nerve injury + 0.9% NaCl), and ceftriaxone (sciatic nerve injury + 50 mg/kg/day ceftriaxone) groups. Treatments were administered intraperitoneally for 12 weeks. Functional recovery was assessed using inclined plane tests and electromyography. Sciatic nerve regeneration was evaluated via histology, nerve growth factor (NGF) immunohistochemistry, and enzyme-linked immunosorbent assay.

**Results:**

Compared to the control group, inclined plane test scores, nerve action potentials, NGF expression, axon counts, and diameters were diminished in both injury groups (*p* 0.001). However, these parameters were significantly improved in the ceftriaxone group compared to the vehicle group (*p* 0.001). Increased fibrosis was observed in the ceftriaxone group.

**Conclusion:**

Ceftriaxone demonstrates potential as a pharmacological agent for PNI recovery by enhancing nerve regeneration and functional outcomes. Further studies are warranted to elucidate its mechanisms.

## Introduction

Peripheral nerve cells form the peripheral nervous system’s basic components that extend from the central nervous system to muscles, organs, and tissues^1^. Peripheral nerve injury (PNI) is a significant health issue that can lead to the loss of sensory and motor functions, often accompanied by chronic pain^2^. The causes of such PNIs include trauma, surgical complications, and other factors such as diabetes and autoimmune disorders^2^. PNIs can occur due to various insults, most commonly due to trauma, surgical complications, compression, diseases, exposure to toxins, and drug-induced complications.

Various techniques are performed to repair or reconstruct the peripheral nerves upon injury. Several minor nerve injuries can heal over time with physical therapy to maintain joint flexibility and muscle strength in the affected area^2^. On the other hand, the pain caused by the damage can be treated with several medications, including non-steroidal anti-inflammatory drugs, opioids, and neuropathic pain medications like gabapentin and pregabalin^3^. In more severe injuries, modalities including end-to-end repair, autologous nerve grafting, nerve transfer, and nerve-guided placement can be applied^4^–^6^. Besides, several other methods currently in the preclinical trial phase are stem cell therapy^7^ and the administration of several growth factors^8^,^9^.

PNI research studies various promising molecules with different clinical applications^9^,^10^. Ceftriaxone is a third-generation antibiotic in the cephalosporin group widely used to treat many bacterial infections^11^. It has high activity against gram-negative bacteria and is effective against some gram-positive bacteria^12^. The anti-inflammatory^13^, neuroprotective^14^,^15^, antiallodynic, and antihyperalgesic^16^ effects of ceftriaxone have also been previously reported.

Ceftriaxone has exhibited neuroprotective effects by diminishing excitotoxicity and inflammation. Ceftriaxone treatment in experimental models of traumatic brain injury decreased cerebral edema, enhanced cognitive performance, and lowered proinflammatory cytokine levels. It also enhanced the generation of glutamate transporter-1 (GLT-1), essential for glutamate clearance and mitigating excitotoxic damage^17^–^19^. Ceftriaxone alleviates neuropathic pain by upregulating GLT-1, diminishing spinal astrocyte activation and neuronal hyperexcitability. This phenomenon has been reported in models of radicular pain and chronic constriction nerve injury, indicating its potential in managing chronic pain disorders^20^,^21^. Moreover, ceftriaxone has demonstrated potential in enhancing functional recovery following spinal cord damage, surpassing monocyte therapy in animal settings^22^.

Rat models of sciatic nerve injury are commonly used to study PNI and regeneration^23^. The primary reason for this is the accessibility of the sciatic nerve and its size, which allows for easy manipulation and evaluation. In this study, we aimed to investigate the effect of ceftriaxone administration after PNI on nerve healing. In this context, the effect of ceftriaxone on nerve healing has been studied electromyographically (EMG), histologically, and biochemically in rats with sciatic nerve damage.

## Methods

### Animal housing and ethical approval

The experimental procedures were conducted at the Experimental Animal Center of T. C. Demiroğlu Bilim University, Istanbul, Turkey. A total of 30 adult male Wistar rats (weighing 200–210 g) was housed under standard laboratory conditions (12-hour light/dark cycle, 22 ± 2°C temperature) with *ad libitum* access to standard rat chow and tap water.

The study was approved by the Local Experimental Animal Research Ethics Committee (Approval No.: 22230510021, on September 23, 2022).

### Group allocation and drug administration

The animals were randomly allocated into three groups:

- Control group (n = 10): no surgical or pharmacological intervention;
- Saline group (n = 10): received 0.9% NaCl intraperitoneally at 1 mL/kg/day during the intervention period;
- Treatment group (n = 10): received 50 mg/kg/day ceftriaxone (IESEF^®^, 1 G) intraperitoneally.

**Figure 1:**
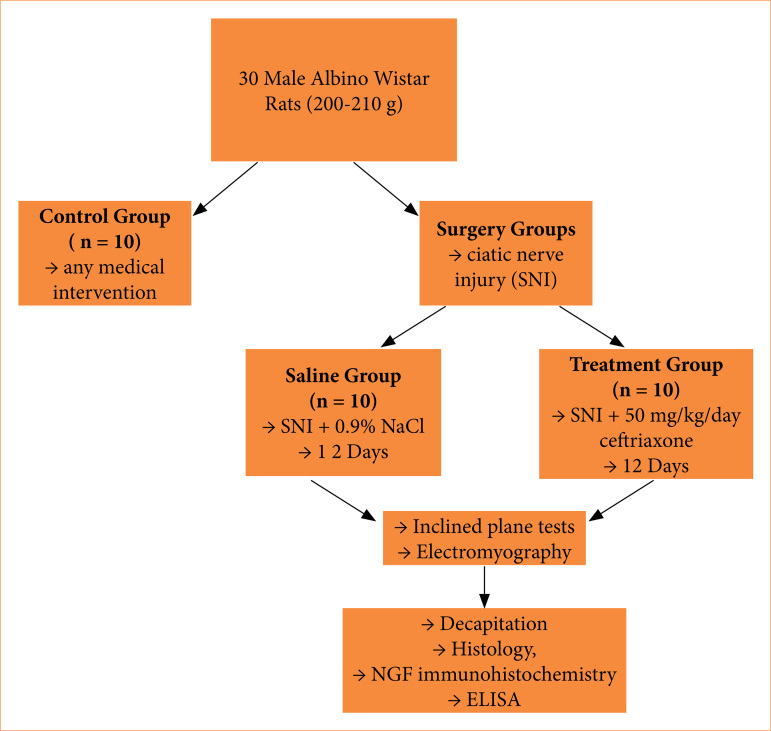
Workflow summary of the study.

Source: Elaborated by the authors.

The treatment continued for 12 weeks (Fig. 1).

### Surgical procedure on sciatic nerves

Rats assigned to the surgical groups were anesthetized with intraperitoneal injections of ketamine hydrochloride (75 mg/kg; Ketasol, Richterpharma AG, Austria) and xylazine hydrochloride (10 mg/kg; Rompun, Bayer, Germany). Aseptic surgical techniques were used to expose and transect the sciatic nerves bilaterally, 1.5 cm proximal to the trifurcation point. Nerve ends were sutured using three epineural 9-0 Ethilon sutures (Ethicon), and skin closure was achieved with 3-0 Vicryl sutures. Postoperatively, the animals were monitored until complete recovery.

### Evaluation of motor function

Motor performance was evaluated using the inclined plane test. Rats were placed lengthwise on a plane initially set at an angle of 10°. The angle was increased incrementally until each rat could maintain its posture for 5 seconds. This was repeated three times per animal, and the average angle was recorded as the motor score.

### Electrophysiological recordings

At the end of the study, rats were anesthetized using ketamine hydrochloride (Alfamine, 80 mg/kg) and xylazine (Alfazyne, 10 mg/kg). EMG responses were recorded bilaterally. Sciatic nerves were stimulated supramaximally at the sciatic notch using a 10 V, 0.05 ms pulse at 1 Hz. Compound muscle action potentials (CMAPs) were recorded from the interosseous muscles via platinum unipolar electrodes. Parameters such as latency and amplitude were analyzed with Biopac Student Lab Pro 3.6.7 software. Body temperature was maintained between 36–37°C during recordings.

### Sacrifice and sample collection

At the end of the 12 weeks, rats were euthanized by cervical dislocation under deep anesthesia (Xylazine 50 mg/kg and Ketamine 100 mg/kg). Blood was collected via cardiac puncture. Sciatic nerves were harvested for biochemical and immunohistochemical analyses.

### Histological and immunohistochemical analyses

The distal sciatic nerve segments (10 mm from the transection site) were fixed in 4% paraformaldehyde, embedded in paraffin, and sectioned into 5-µm slices. Hematoxylin and eosin staining were used for general histological evaluation. Fibrosis was quantified by counting cells in five randomly selected regions per section.

For immunohistochemistry, sections were incubated with 10% H_2_O_2_ to inhibit endogenous peroxidase, blocked with normal goat serum, and incubated overnight at 4°C with anti-nerve growth factor (NGF) antibody (Santa Cruz Biotechnology; 1/100). Detection was performed using DAB and the Histostain-Plus Bulk kit. Images were captured with an Olympus BX51 microscope and C-5050 camera. Schwann cells and NGF-positive axons were counted in six sections per group using 20x magnification in randomly selected fields.

### Measurement of nerve growth factor levels by ELISA

Sciatic nerve segments were rapidly dissected and stored at -20°C. Tissues were homogenized in phosphate-buffered saline (pH 7.4), centrifuged at 5,000 × g for 15 minutes, and collected supernatants. Protein concentrations were measured using the Bradford method^24^. NGF levels were quantified using a rat-specific enzyme-linked immunosorbent assay (ELISA) kit (triplicate measurements per sample) and read using a MultiscanGo microplate reader (Thermo Fisher Scientific).

### Statistical analysis

Data were analyzed using Statistical Packages for Social Sciences 22.0 software (IBM, Armonk, NY, United States of America). Categorical data are presented as frequency and percentages and analyzed using the χ^2^ test. Measurement data are displayed as mean ± standard error of the mean (SEM), and the parametric component was compared using analysis of variance (ANOVA) and Student’s t-test. The non-parametric component was compared using the Mann-Whitney’s U test. Following a normality test, a Student’s t-test was used for comparison. *P* < 0.05 was considered statistically significant.

## Results

Table 1 shows that the control group had considerably higher inclined plane scores (*p* < 0.001), whereas the ceftriaxone group had significantly higher ratings (*p* < 0.001) when compared to the saline group.

**Table 1:** Inclined plane scores electromyographic (EMG) compound muscle action potentials (CMAP) latencies, EMG CMAP amplitudes of the groups. Data are expressed as mean ± standard error of the mean.

	Control	Saline	Ceftriaxone
Inclined plane score (°)	87.4 ± 0.8	36.3 ± 1.8*	65.7 ± 1.4* ^#^
EMG CMAP latency (ms)	2.34 ± 0.07	3.59 ± 0.14*	3.43 ± 0.15*
EMG CMAP amplitude (mV)	12.5 ± 0.6	1.8 ± 0.25*	5.03 ± 0.5* ^#^

* *p* < 0.0001, compared to control group;

# *p* < 0.0001, compared to vehicle group.

Source: Elaborated by the authors.

**Figure 2:**
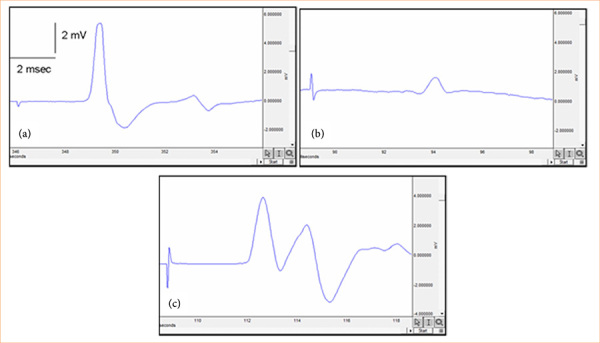
Representative electromyographic signals of the groups. **(a)** Control group, **(b)** saline group, and **(c)** ceftriaxone group.

Source: Elaborated by the authors.

Both the saline and ceftriaxone groups had substantially longer EMG CMAP latency than the control group (*p* < 0.001; Table 1). The control group had a substantially higher EMG CMAP amplitude (*p* < 0.001; Table 1; Fig. 2), while the saline and ceftriaxone groups had a significantly lower amplitude. Table 1 and Fig. 2 show that the EMG CMAP amplitude was considerably more significant in the ceftriaxone group than in the saline group *p*p < 0.001).

**Figure 3:**
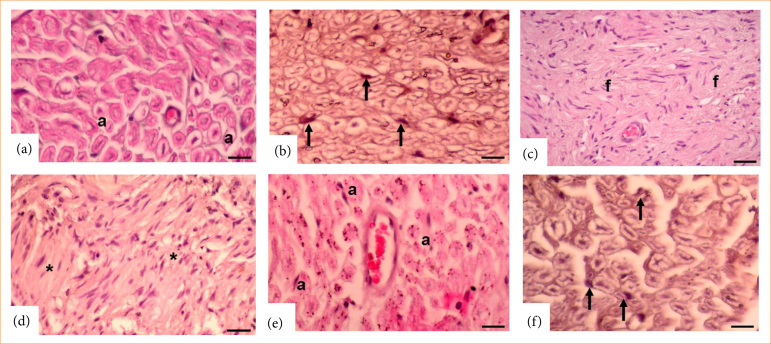
Hematoxylin and eosin and nerve growth factor (NGF) immunostaining. (**a** and **b**) Control group. a: normal axon and arrow: Schwann cell. (**c** and **d**) Saline group. Increased fibrosis. f: Greatly reduced axon, Schwann cell, and NGF immunoreactivity (asterisk). (**e** and **f**) Ceftriaxone group. a: increased axon, Schwann cell, and arrow: NGF immunoreactivity (Magnification = 20x).

Source: Elaborated by the authors.

Compared to the control group, the groups treated with saline or ceftriaxone showed significantly decreased levels of NGF immunoreactivity in Schwann cells (p < 0.0001; Table 2 and Fig. 3). Table 2 and Fig. 3 show that the ceftriaxone group had substantially increased NGF immunoreactivity in Schwann cells compared to the saline group (*p* < 0.0001). Overall, there was a substantial decrease in the number of axons in the control group, in both the saline and ceftriaxone groups (*p* < 0.0001; Table 2 and Fig. 3), but a significant increase in the ceftriaxone group relative to the saline group (*p* < 0.001; Table 2 and Fig. 3).

**Table 2:** Nerve growth factor (NGF) immunoreactivity in Schwann cells (%), total axon number, average axon diameter (µm), and fibrosis score (%) of the groups. Data are expressed as mean ± standard error of the mean.

	Control	Saline	Ceftriaxone
NGF immunoreactivity (NGF) (%)	75.6 ± 4.2	9.9 ± 1.2*	57.2 ± 2.9*^#^
Total number of axons	285.9 ± 24.5	23.6 ± 5.3*	121.7 ± 10.3*^$^
Axon diameter (µm)	3.29 ± 0.17	1.75 ± 0.18*	2.8 ± 0.15^$^
Fibrosis score (%)	1.6 ± 0.2	71.1 ± 3.3*	13.9 ± 1.2*^$^

* *p* < 0.0001, compared to control group;

$ *p* < 0.001, compared to vehicle group;

# *p* < 0.0001, compared to vehicle group.

Source: Elaborated by the authors.

The control group had a considerably larger axon diameter than the saline group (*p* < 0.001; Table 2 and Fig. 3), whereas the ceftriaxone group had a significantly larger axon diameter than the saline group (*p* < 0.001; Table 2 and Fig. 3).

The control group had considerably lower levels of fibrosis (*p* < 0.001; Table 2 and Fig. 3), whereas the ceftriaxone group had significantly lower levels (*p* < 0.001; Table 2 and Fig. 3), in comparison to the saline and ceftriaxone groups.

The levels of NGF were noticeably lower in the group that was transported in a saline when contrasted with the control group (*p* < 0.01; Table 3). Despite the lack of statistical significance among the other groups, NGF levels were approximately twice as high in the ceftriaxone group as in the saline group (p = 0.09; Table 3).

**Table 3:** Nerve growth factor (NGF) levels in the groups. Data are expressed as mean ± standard error of the mean.

	Control	Saline	Ceftriaxone
NGF levels in the sciatic nerve (pg/mg)	25.5 ± 2.7	14.6 ± 1.5*	20.9 ± 1.6

* *p* < 0.01 compared to control group.

Source: Elaborated by the authors.

## Discussion

Traditional treatment methods for PNI include physical therapy, pain management, and surgery in severe cases. At the same time, emerging research indicates that pharmacological agents may play a promising role in facilitating nerve healing^25^. This study showed that ceftriaxone administration after sciatic nerve injury showed partial improvement in EMG parameters and muscle strength, and neuronal markers in rats were examined.

Nerve injuries can affect an animal’s strength and balance on the inclined plane^26^. The animal’s maximum plane angle increases as nerve healing progresses, indicating improved function. Gurkan et al.^27^ found that oxytocin improves PNI rats’ inclined plane scores. This study also found that ceftriaxone improved the maximum angle rats could maintain on the inclined plane compared to rats with only sciatic nerve damage. Ceftriaxone may improve nerve healing and motor function. Axons and myelin sheaths can be damaged when a peripheral nerve like the sciatic nerve is injured^27^, and axon number and diameter can affect functional outcomes.

Functional recovery is usually better with more axons and diameters^28^,^29^. In this study, the saline group had fewer sciatic nerve axons and smaller diameters than the control group. While the ceftriaxone group did not fully recover, their parameters were significantly higher than the saline group. Through pathway modulation, digoxin^30^, folic acid^31^, and gallic acid^32^ aid nerve healing. In rat Parkinson’s disease models, ceftriaxone improved motor deficits^32^,^33^ and nigral oxidative damage^33^. Ceftriaxone improves motor function, neuronal apoptosis, and neuroinflammation in rat traumatic brain injury models^33^,^34^.

Few studies have examined ceftriaxone’s effects on fibrosis. In rats, Abdel-Daim et al.^35^ found that ceftriaxone significantly reduced transforming growth factor (TGF)-β 35 levels in cisplatin-induced renal fibrosis. Logan et al.^36^ found that TGF-β contributes to scar formation and fibrosis. In this study, saline animals had significantly higher fibrosis scores than the control group. Though not completely normalized, these parameters in the ceftriaxone group were significantly lower than those in the saline group. These results may be due to lower TGF-β levels.

Ceftriaxone has demonstrated the ability to inhibit signs of neuronal autophagy, which may be harmful after traumatic brain damage^37^. Conversely, ceftriaxone can reduce oxidative stress indicators with other medicines, such as selenium, offering additional protection to neurons against injury-induced damage^38^. Furthermore, it diminishes the expression of proinflammatory cytokines, including interleukin-1β, interferon-γ, and tumor necrosis factor-α, which are enhanced after nerve injury^18^,^38^. The diminishment of inflammation enhances its neuroprotective properties. Ceftriaxone alleviates glutamate excitotoxicity by enhancing GLT-1 expression, a significant factor in neuronal damage post-nerve injury^39^.

In this study, ceftriaxone increased CMAP amplitude but not latency in animals with sciatic nerve damage. Latency depends on the fastest distal fibers, while CMAP amplitude shows the number of motor fibers activated upon stimulation. Due to muscle innervation and axonal loss, sciatic nerve injury often decreases CMAP amplitude^40^. Numerous studies have examined ceftriaxone’s neuroprotective effects, separate from its antibacterial effects^41^–^43^. Ceftriaxone increases GLT-1 expression, which removes glutamate from the synaptic cleft^44^. Increasing GLT-1 expression, ceftriaxone may limit this damage and protect nerves from injury^45^. Nerve injuries often cause local inflammation, which worsens the damage. Ceftriaxone reduces inflammation, which reduces nerve damage and improves nerve regeneration, increasing CMAP amplitude.

Yalçın et al.^46^ demonstrated that the activation of autophagy and anti-inflammatory mechanisms are essential in improving diabetic neuropathy, underscoring the therapeutic potential of targeting these pathways in nerve injury^47^. The findings indicate that reducing inflammation and enhancing cellular homeostasis may facilitate nerve regeneration and functional recovery. In our study, the neuroprotective effects of ceftriaxone may be partially due to its capacity to modulate inflammation and cellular stress responses, similar to the mechanisms observed with rilmenidine. The similarities among these studies indicate that pharmacological agents possessing anti-inflammatory and autophagy-modulating characteristics may enhance axonal regeneration and motor function recovery after peripheral nerve injury.

A study by Quarta et al.^48^ claims that NGF protects neurons from damage and nerve regeneration following injury. Similarly, Ma et al.^49^ found that NGF promotes the repair of peripheral nerves by inducing differentiation of neuronal cells and enhancing neurite growth, thereby enhancing recovery after PNIs such as sciatic nerve damage. In this study, the ceftriaxone group showed a substantial rise in NGF immunoreactivity in Schwann cells, whereas the saline group showed a considerable drop. Both the control and ceftriaxone groups had greater NGF levels than the saline group, but there was no statistically significant difference. These suggest that ceftriaxone may improve the decreased levels of NGF by sciatic nerve injury, contributing to improved healing.

### Limitations

While this study demonstrates the beneficial effects of ceftriaxone in a rat model of sciatic nerve injury, the results may not fully translate the complexity of human nerve regeneration. Although animal models are essential for preclinical research, further human studies are necessary to confirm these findings. The long-term effects and potential adverse outcomes of ceftriaxone therapy were not assessed. Future clinical trials are needed to evaluate human subjects’ safety, efficacy, and optimal treatment protocols.

## Conclusion

Study results suggest that ceftriaxone can contribute to nerve healing, but it may not be sufficient for complete functional recovery in PNIs. Moreover, in addition to ceftriaxone, local administration of several other compounds might be beneficial in achieving full functional recovery from PNIs.

## Data Availability

All data sets were generated or analyzed in the current study.
